# A systematic review of comprehensive Robotic-assisted surgical (RAS) curricula

**DOI:** 10.1007/s11701-025-02962-6

**Published:** 2025-12-29

**Authors:** Anna K. Kieslich, Ruari Jardine, Hussain Ibrahim, Areeg Calvert, Kenneth G. Walker, Kim A. Walker, Angus J. M. Watson

**Affiliations:** 1 NHS Highland, Raigmore Hospital, Old Perth Road, Inverness, IV2 3UJ Scotland, UK; 2 Centre for Healthcare Education Research and Innovation (CHERI), University of Aberdeen, Aberdeen, Scotland, UK; 3 University of Aberdeen, Aberdeen, AB24 3FX Scotland United Kingdom; 4 Royal College of Surgeons of Edinburgh, Nicolson Street, Edinburgh, EH8 9DW Scotland United Kingdom

**Keywords:** Robotic assisted surgery, Curriculum, Surgical training, Skill, Simulation systematic review

## Abstract

Robotic assisted surgery (RAS) is being increasingly used for minimal access surgery. There has been a growing interest in developing curricula for RAS training. We performed a systematic review of comprehensive RAS curricula. Methods: A systematic review of RAS curricula evidence and teaching was conducted on the platforms Medline, PubMed, Embase, CINAHL and PsycINFO combining robotic surgery with surgical training in February 2024 and updated in June 2025. After removal of duplicates 10,002 references were returned. Screening using the PICO framework for eligibility identified 175 relevant studies, out of which 39 studies reported on 29 comprehensive RAS curricula. Data were extracted and analysed using a predeveloped extraction tool. Analysis focused on common curricular elements and supporting evidence for curricula success according to Kirkpatrick’s levels of evidence. Study quality was assessed using the MMERSQI (Modified Medical Education Research Study Quality Instrument) criteria. PROSPERO CRD42024566778. All 29 curricula included simulation training. The nature and volume of simulation training and mentorship varied widely across curricula. Objective assessment and non-technical skills training (NTS) were not uniformly included. Surgical societies outlined curricula with proficiency-based progression, modular intraoperative training and objective assessment. All studies found a benefit from curriculum implementation, but the methodological quality of evidence was low. Conclusion: A standardised RAS curriculum is feasible and beneficial and surgical societies have outlined RAS curricula which could be implemented locally. However, not all current comprehensive RAS curricular are grounded in the relevant educational frameworks. Further research supporting the implementation and evolution of RAS curricula is necessary.

**Supplementary Information:**

The online version contains supplementary material available at 10.1007/s11701-025-02962-6.

## Introduction

Robotic assisted surgery (RAS) was introduced as an advancement of minimally invasive surgery. RAS enables the performance of a wider range of operations utilising better ergonomics, improved visualisation, and a greater range of instrument movement compared to laparoscopic surgery [1]. Surgeons need to acquire a unique skill set to enable them to operate the surgical robot and this has led to the development of ‘novel’ training methods [2]. With an exponential increase in uptake [3], robotic surgery has been seen as having a negative impact on surgical trainees. Concerns regarding the limited access to RAS training as well as reduced exposure to other operative modalities have been reported [4]. Therefore, there is an increasing demand for the formal incorporation of RAS training into the surgical training curriculum [4–6]. 

Ahead of curricula development and implementation, the evaluation of previously published curricula is an important aspect of identifying the ideal approach to meet the learning needs [10, 11] 

**Fig. 1:**
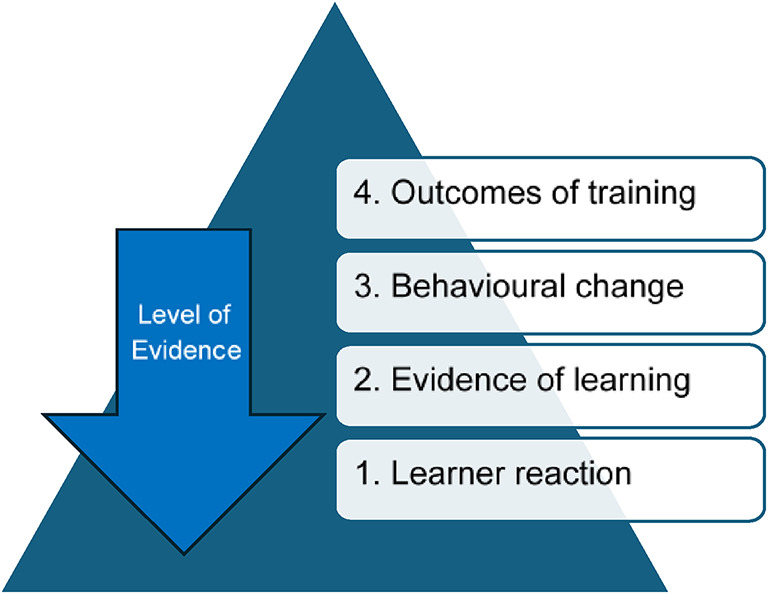
Levels of evidence attributable to a training program adapted from Tamkin et al. [13]

Kirkpatrick’s levels of evidence were used to examine the quality of evidence available for RAS curricula assessment. To evaluate the attributable effect of teaching programs, Kirkpatrick et al. outlined four levels of evidence. The ascending levels of attributable benefit of a teaching program are (1) how well trainees received a program such as trainee feedback regarding a course or subjective levels of confidence, (2) measured learning, this is quantifiable evidence that learning has occurred attributable to the program3) change in behaviour attributable to the program, this will require evidence of application of the learned skill in practice 4) outcomes of training, such as reduced cost or higher quality of outcomes delivered, which can be linked to the training. The importance of an adequate before-and-after assessment approach with a control group and statistical considerations were highlighted as key to this evaluation [12]. (Fig. 1).

The levels of evidence were initially developed for corporate management have been widely used since its publication in 1959. Further levels on societal impact and on return of investment have been discussed more recently [13]. Kirkpatrick levels can be applied to RAS training. Level 2 equates to RAS skills and can be assessed with validated scoring systems during simulation [14]. Level 3 can be assessed by the uptake and delivery of RAS procedures in the operating theatre (number of cases) and intraoperative evaluation scores such as GEARS (Global Evaluative Assessment of Robotic Skills) [15], OSATS (Objective structures assessment of technical skills) [16] and NOTSS (Non-Technical skills for surgeons) [17, 18]. Level 4 equates to operative outcomes after RAS (including complications and metrics like length of stay). Level 5 can be translated into impact on waiting lists and measured health economics.

In the RAS educational literature, the term ‘curriculum’ refers to a wide range of teaching interventions. Publications vary from a description of a selection of virtual reality (VR) simulator tasks to a comprehensive teaching syllabus taking a robotic novice to independent operating [19, 20]. For this review, we have only considered RAS curricula which include the whole teaching syllabus, from RAS novice to independent practice.

The aim of this publication is to systematically review the content and published outcomes of existing comprehensive RAS curricula. The review will inform the development of a platform-agnostic RAS training curriculum for surgical trainees.

## Methods

### Search and sources

**Fig. 2:**
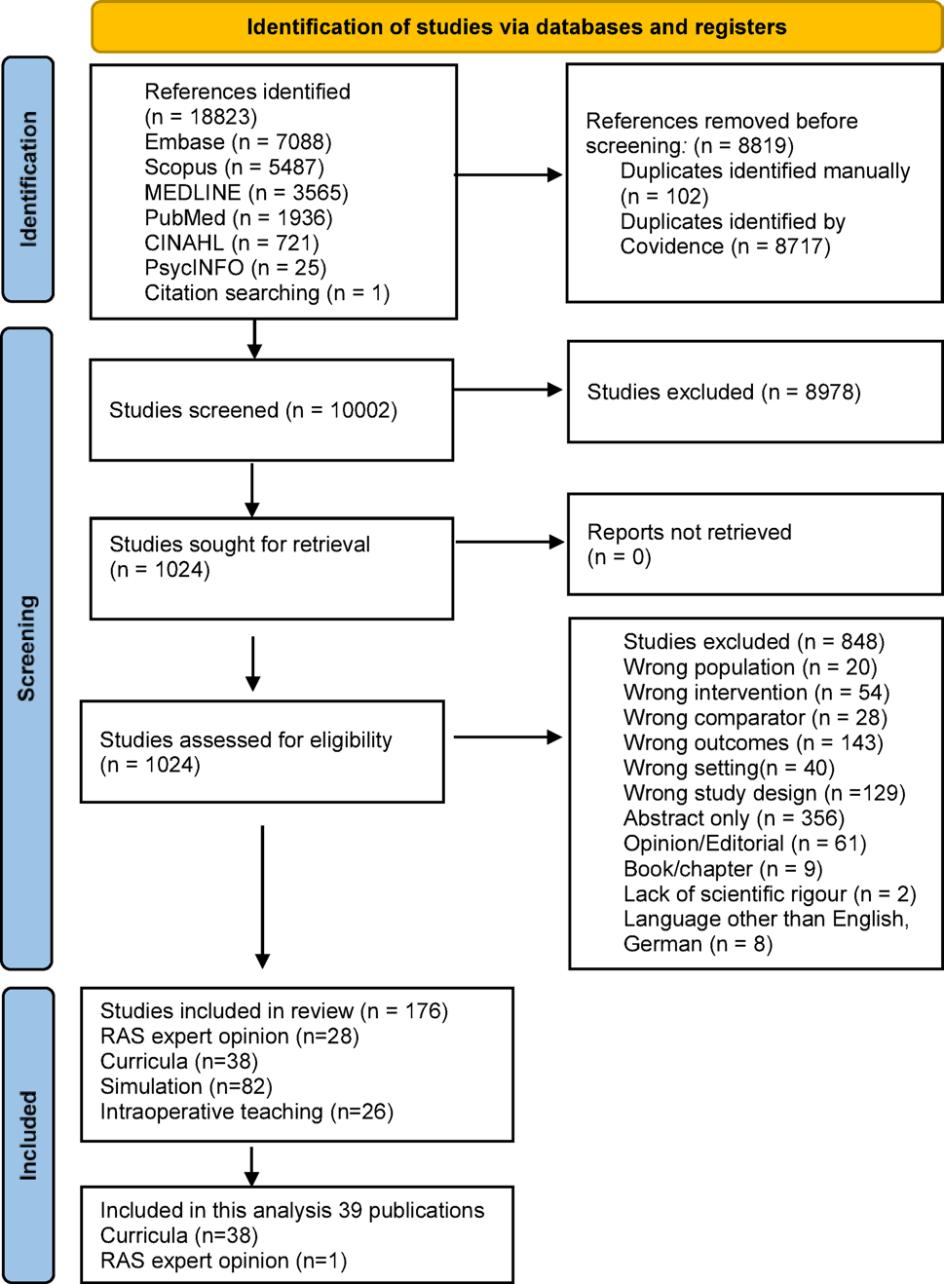
Prisma Flow Diagram

Our methods have been published previously [21]. This systematic review follows the PRISMA (Preferred Reporting Items for Systematic Review and Meta Analysis 2020) Guidelines [22]. See Fig. 2 for PRISMA Flow Chart and Appendix 1 for PRISMA checklist. We used Covidence systematic review software (Veritas Health Innovation, Melbourne, Australia, available at www.covidence.org) for title and abstract screening, full-text review and data extraction. Systematic platform-adapted Boolean literature searches, built with the aid of an information consultant and a librarian, combining RAS with training were conducted across six different databases (Medline, PubMed, Embase, Scopus, Cinahl, Psych Info) in English and German from 1997, when the first RAS operations were performed, to the current day (2025). Even though PubMed contains Medline, a separate Medline search resulted in 512 additional articles, which we included in the screening. See Appendix 1 for platform specific searches.

The searches were conducted between 17/01/2024 and 06/02/2024 after several scoping searches and updated on 20/06/2025. Reference chaining was used to ensure data saturation.

The search yielded 18823 references, with 10,001 studies after exclusion of duplicates. Title and abstract screening excluded 8990 studies as irrelevant. Out of 1010 full-text studies assessed for eligibility, 175 studies met the inclusion criteria. 39 publications described a comprehensive RAS curriculum. (PRISMA Flow-Chart Fig. 2) The review was registered on PROSPERO under ID Number CRD42024566778 on 02/10/2024.

### Study selection

We defined study eligibility criteria using the PICO (population, intervention, comparator, outcomes) framework (Table 1).

**Table 1:** PICO Inclusion/Exclusion criteria

Eligibility Framework	Inclusion	Exclusion
Population	Surgeons	Medical students, other health professionals
Intervention	Curriculum and teaching elements	Questionnaires, ultrashort interventions (single trial on simulator/one day course)
Comparator	Curricula, training methodology	Learning curve
Outcomes	Completion/engagement in curriculum, meeting predefined standard, patient outcomes	Outcomes other than teaching/training of robotic surgery e.g. simulator validation, feasibility of teaching, quality assessment mechanisms (crowd-based video review and robotic surgery assessment scores)
Setting	General Surgery, Urology, Gynaecology, cardiothoracic surgery	Orthopaedic surgery, Ear Nose and Throat (ENT) Surgery, Neurosurgery
Publications	Peer-reviewed original publications	Abstracts, Books, Literature Reviews, Questionnaires, Editorials, single surgeon case series

We focused the setting on RAS in the specialties of general surgery, gynaecology, urology and cardiothoracic surgery. These specialties have a comparable operative field in a confined cavity and use the Da Vinci platform (Intuitive, Sunnyvale CA, USA), as well as the emerging surgical robots such as Versius (CMR Cambridge, UK) and Hugo (Medtronic, Minneapolis MN, USA).

Although initially included in the search, we excluded articles on learning curve as they often were presented as a case series and mostly did not describe the method of learning or teaching. AK plus one of RJ, HI, AC or AW did the title and abstract screening. AK plus one of RJ or AW did the full text review. Conflicts were resolved by discussion. Data extraction and analysis was done by one reviewer (AK) and cross-checked by another (AW) to reduce bias.

### Data extraction and analysis

The papers included in the systematic review were heterogenous. We analysed studies concerning comprehensive RAS curricula with comparable methodology and outcomes together. Here we analysed studies concerning comprehensive RAS curricula. To increase the understanding of curriculum content, we considered any additional information, cited in publications.

Data were extracted systematically, using a previously developed extraction tool adapted for the study methodology. We examined common curriculum elements and evidence of outcomes reported. Because of wide heterogeneity of interventions and comparators presented in the publications, a metanalysis was not feasible.

The quality of studies was assessed using the Modified Medical Education Research Study Quality Instrument (MMERSQI)tool with a minimum of 23.5 and a maximum score of 100 [23]. The modified MERSQI scoring does not differentiate between descriptive statistics or simple inferential statistics. Points are doubled when modelling is used. The distinction between inferential statistics and complex modelling is not clearly defined in the MMERSQI publication. We defined complex modelling as using any statistical test, which could be seen as a model using graphs and/or a deeper examination of the data than description or percentage. We used this low threshold to enable a quality distinction between studies. The potentially ‘artificial’ increase of the MMERSQI score for these studies was 4 points in total. Studies that used a previously validated assessment method like GEARS or a validated simulator score were scored YES for validity of assessments, even if the validity was not provided in the publication itself. (Table 2).

**Table 2:** Summary table of curricular content, study methodology and outcomes

Curriculum YEAR Country Specialty Duration	Knowledge delivery	Simulation	Operative Practice	Educational concept/ Assessment	Participants No Type	Aim/ Method	Comparators/ Outcome measures	Outcome	Funding/ MMERSQI
Mirheydar 2009 USA Urology -Extended proctorship [24, 25]	Intuitive surgical training course, 2 × 8 h sessions	Porcine lab	Assist proctor in 5/6 RALP, gradual increasing console surgeons’ manoeuvres, easier to difficult steps	Step wise increase of operative steps by difficulty -trainees required extended proctoring (1 year)	9 Urologist, 4 training postgraduate Urologists, 3 fellows, 2 junior Urology faculty	Assess the impact of RAS proctorship up to 32 months of follow-up/ Observational	Comparing trainees in program -number of cases proctored, -take-rate, - number of cases performed 2005–2007	Mean number of proctoring 20.1 cases (range, 5 to 40) until independence. Trainees required 1 year of proctoring. 6 of 9 urologists undertook training with a partner. 100% take-rate.	No funding 46
Galloway 2012 USA Gynaecology 8-week course in 4-year curriculum [25]	Lectures and one online tutorial; NTS: decision-making skills, taught during all steps of curriculum	Simulation, Dry and Wet Lab, inanimate and on chicken-leg including suturing 4 × 4 h	-Perform any portion of surgery during assistance as allowed by attending - Y3/Y4 after 4 weeks of 8-week training operate cases from their own clinic with assistance	Curriculum includes knowledge, basic skills, experience, decision making, performance and evaluation: Evaluation after each surgery, -goal of 5 independent surgeries and 50 assists - Global ACGME^a^ evaluation at completion of rotation	24 residents Post Graduate Year (PGY)1–4, operative PGY3-4	Formally incorporate RAS GYN surgery into standard 4-year curriculum/ Retrospective observational	Pre- post skill evaluation/ -simulator times -number of cases bedside/console	Significant improvement of simulator times Y3-4 more “skill” than Y1-2; Operative Y3/4: bedside 103, console 25 surgeries in total	Not declared 45
Seder 2013 USA Thoracic surgery 3 years [26]	Individual phase 1: Industry provided online modules	Proctored phase 2: Intuitive led dry-lab practicum, mentored cadaveric course	ProctoredClincal phase 3: Case observation and bedside assist, console at mentors’ discretion	Mentor approval of all stages (dry lab, cadaver simulation and operative training) before progressing to next phase	3 Residents and 1 surgeon	Report structured, competency-based pathway/ Retrospective observational	Patient and operative variables, take rate 2008–2012	79 cases in 3 years, 20% complication rate, fewer major complications in later cases, 100% uptake of RAS in participating surgeons	No funding 47
**EAU/ERUS** ^b^ Volpe 2015 EUROPE (Belgium) Urology 2015 6 months fellowship [19, 27]	ERUS E-learning module	1week of structured, simulation-based laboratory training VR, DRY, WET	Modular training progressive, proficiency-based training through surgical steps with increasing levels of complexity	Modular/proficiency based. Full RARP procedure assessed by mentors GEARS + RARP^c^ score, independent blinded video review	10; 3 resident, 5 fellow, 2 Staff	Feasibility, acceptability, face validity, and educational impact of RARP curriculum /Prospective observational	Pre/post skills assessment (baseline, week 4, end of curriculum)/mentor GEARS and RARP^c^ procedure score, video review score	-dVSS score significantly increased over time for all. GEARS good to excellent 80–100% of trainees. −8 (80%) able to perform a RARP independently, safely, and efficiently by mentors. 3(30%) able to perform a complex RARP independently, safely, effectively by video. 8 out of 10 surgeons considered safe (Score > 10) for every step by video review. 18 (14–36) RARP performed in 8 weeks	No funding 80
**ERUS/EAU** Diamand 2023 Belgium Urology [28]					1 Novice urologist	Investigate safety and efficacy of RAS Cystectomy curriculum/ Prospective observation	-Trainee vs. mentor - operative steps/ -perioperative clinical data 2021–2022	209 Steps (21 cases) 168 (80%) attempted, 125 (60%) performed. Equal: Bloods loss, operative quality, complications. Significantly longer operative time.	ERUS 70.5
Winder 2016, USA General surgery 5-year residency [29]	Online modules, reading assignments NTS: Trouble shooting Intuitive surgical, Sunnyvale, USA program	Complete 10 h of VR simulation until trainees feel comfortable with a task	10 Bedside cases until sitting at the console, operative console training as opportunity arises	Time-based, self- reported, Central logbook maintained, trainees self-report their progress through the curriculum	20 Residents	Share initial experience of development and implementation of RAS surgical curriculum /Retrospective observational	-Self-reported completion of pre-clinical curriculum, -residents self-reported participation in RAS 2014–2015	All 20 residents reported completion of online/bedside curriculum, Simulation by residents on busy RAS services. 13/20 (65%) sat at console for at least one case, 2/6 (33.3%) PGY2, 4/5 (80%), PGY3, 5/5 (100%) PGY4, 2/4 (50%) PGY5.	No funding27.5
**UPMC CGSO** ^d^ **University of** **Pittsburgh Curriculum** 2017 USA General surgery 2-year complex Oncology UGI^e^ Fellowship [30, 31]	Video library	1) Pretest, 2-week VR curriculum, post-test 2) Bio tissue drills video-recorded and graded by independent observer	3) Intraoperative coaching 4) review of surgical videos in a digital video library 5) ongoing clinical assessment in live operating room cases	Mastery/Proficiency based VR score > 90 green checks or > 10 attempts. OSATS bio tissue. Video review, exact method of final assessment not detailed					
**UPMC** **VR Simulation** Hogg et al. 2017 USA UGI Duration see above [32]		VR curriculum			17 General surgical oncology fellows	Content/predictive validity RAS simulator curriculum/prospective observational	Pretest/post-test of participating fellows/ VR Simulator scores on ‘difficult tasks’, inanimate tasks video OSATS July 2013-Dec 2014	Significant improvement on VR scores for all fellows, significant improvement on inanimate video scores for most fellows (15/16)	Intuitive surgical, Sunnyvale, CA, USA 65
**UPMC** **Pancreatic bio tissue** Tam et al. 2017 USA UGI Duration see above [33]					14 Upper GI Complex Oncology fellows, 3 attendings	Feasibility and validity of bio tissue curriculum / Prospective observational,	Pre-test post-test skill evaluation/Time to completion, errors, -OSATS score	Face validity and OSATS benchmarks established by 3 attendings Increased OSATS, decreased errors and decreased time with increased number of attempts	Intuitive surgical, Sunnyvale, CA, USA 73
**UPMC** **evolution** Knab et al. 2018 USA UGI Duration see above [30]					30 fellows	Describe evolution and outcomes of a proficiency-based robotic training program / Retrospective observational	Review of fellowship portfolios/Number of cases with fellowship participation, Take rate 2013–2017	Fellows on average: 5 h VR curriculum,19 bio tissue anastomoses. Median 33 RAS cases (12–80). Fellows console operating increased over time (*p* = 0.005). -Average% of RPD^f^ steps increased (p\0.011), Number of entire RPD resection increased (*p* = 0.013). 87.5% use RAS post fellowship, 91% HPB^g^ surgery.	No funding/ 71
**UPMC** **Hernia curriculum** Tam et al. 2019 USA UGI Duration not specified [34]		Virtual reality simulation (5–8 h), bio tissue curriculum (1–2 h)	Live OR proctorship during 3 robot hernia cases with expert feedback or video review	Proficiency based curriculum, assessment not specified, bio tissue hernia curriculum analogous to Pittsburgh pancreatic curriculum	16/6 vs. 10 surgeons	Impact of curriculum on clinical outcomes and cost/ Retrospective review, QI project, unrandomized groups, post-test only	-Surgeons taking part in curriculum vs. traditional training -operative time, complications, cost 2015–2017	Significantly lower risk adjusted operative time and cost (20% reduction) for curriculum trainee vs. non-trainee. A typical non-trainee would need approximately 28 cases to match the expected operative time of a typical trainee on their first case.	Veteran affairs salary support/ 76
**UPMC** Rice et al. 2020 USA UGI Duration see above [35]					26 surgeons, 2 early adopters, 2 mentored surgeons, 24 fellows with formal curriculum	Effect of mentorship and curriculum on learning curve/Retrospective review of patient data	Comparing 3 generations of surgeons (early adopters; with mentorship; with curriculum) -perioperative clinical data 2008–2017	Improved operative time, conversions, blood loss lymph nodes and length of stay with subsequent generations. Other factors same. Flattened learning curve.	SAGES/Intuitive surgical 79
**UPMC** Ahmad 2021 Play Peoria USA UGI Duration see above [36]					30 surgical fellows 15 = UPM, 15 = non UPM^h^	Implementation of the UPMC curriculum for trainees in 4 other institutions /Prospective observational	Compliance and pre/post skill UPM vs. non-UPM/ -Simulator Scores - engagement	Little engagement with the curriculum outside UPMC. Mastery associated with time spent on curriculum UPM significantly better on post-test VR scores (only comparing fellows, who completed the curriculum)	Intuitive surgical, Sunnyvale, USA 76
**LAELAPS-3** **based on UPMC** Zwart et al. 2022 Netherlands UGI Duration of training not specified [37]			Opposite to the UPMC curriculum no extended mentoring fellowship, 16 proctoring sessions for 15 surgeons, reported in paper as median of 2 (1–3) session per surgeon		15, surgeons with 5-year experience with pancreatic surgery	Safety and feasibility of a multicentre training program in RPD in the Dutch healthcare setting./ Prospective review	RPD fellows before and after operative time inflection point/ Perioperative clinical outcomes of first independent cases (7 centres/after UPMC curriculum) 2016–2019	3 centres did not reach minimum required number over study period, 2 proctoring sessions/surgeon, operative time inflection point at 22 cases, comparable outcomes before and after inflection point, higher fistula rate than prev. case series	Intuitive, Ethicon, Johnson& Johnson 58.5
**SERGS** ^i^ Rusch et al. 2018 European Society (Germany, France, Sweden, Spain, Belgium, Netherlands) Gynaecology 12–13 months Duration:10 months in-house training [38]	Online modules, didactic in-house training	Virtual in-house training (if available), Dry and Wet Lab training at ORSI (5 days)	In house training moderate to complex gynaecological procedures. NTS: During mentorship phase, mentors were unfamiliar with NOTSS score	Modular curriculum, requiring completion of each module, before moving on to the next; Logbook, video evaluation from another tutor, final approval of SERGS committee	4 Fellows, certified gynaecologists novice in RAS	Feasibility and effectiveness SERGS pilot curriculum /Prospective observational	Progress of curriculum over time -GEARS video scores post-test, -ORSI simulator evaluation pre/post	After 10 months of training all 4 candidates receive GEARS scores of 15 or above all improved simulator scores at ORSI; more open assessment than GEARS was used during mentoring, training not done stepwise	Intuitive Surgical, Inc 74
**APDCRS** ^j^ Martin et al. 2019 Colorectal USA 5-year Residency/duration of study 1 year [39]	Intuitive online modules, 3 surgeon led webinars.	Intuitive in-service and simulation, Advanced course: -spring cadaver course -case log -simulator	Console practice, “modular” stepwise. NTS: Trouble shooting/tips and tricks webinar	Standardise, progress tracking, completion of assessed steps, skills simulator > 90%, 5x console surgeon before cadaver course 10 bedside assists, 20 console cases > 50%	40 out of 93 colorectal residents	Impact of novel case log-system on training /Retrospective observational	Observation of RAS cases logged by residents in one year -procedure type, - number of cases logged 2016–2017	1118 cases logged, 745 (66%) as console surgeon. 26 (65%) of 40 residents performed. 20 or more RAS procedures.	No funding/ 42.5
Moit et al. 2019 USA General surgery 5-year residency Phase 1 (mandatory)PGY1-2 Phase 2 (self-selected) PGY3-5 [40]	Read two assigned articles, complete Intuitive online training, intuitive online videos encouraged	Dry lab with intuitive rep, Intuitive VR simulations	4 bedside assist of 4 cases with 2 faculty, console operating with increasing level of complexity 2 faculty; NTS: Not many formal NTS assessments filled out	Stepwise progression through phases, Simulator exam > 90%, 4 bedside cases, 10 console cases with 2 different faculty, attending will evaluate every case by GEARS assessment, certificate of completion possible for phase 2 residents	18 general surgery residents	Investigate standardised RAS curriculum and evaluation/ Retrospective observational	Progression of GEARS rating over time/cases performed, GEARS	6 out of 18 residents had 5 or more assessments. Positive correlation between performance and experience for all residents. Three residents showed a strong correlation between their overall performance and number of procedures and 3 showed a low correlation. Only one resident applied for certificate of completion.	No Funding 55.5
Mustafa et al. 2019 General surgery USA Duration not specified [41, 42]	Online modules. NTS: Troubleshooting teaching by industry rep.	One-on-one bedside teaching by industry, VR simulation	10 cases bedside assist, begin to sit on the console performing part of the operation with the goal of completing cases on own, 25 cases until evaluation	90% simulator. score, 10 bedside assists (manufacturer recommendation), 30 console cases, evaluation of last 5 cases signed off by staff	Residents, number not specified	Does RAS curriculum enhance mic. Training?/ Retrospective observational	Pre- post curriculum / -operative mode and residents’ involvement of hernia cases 2013–2017	Hernia cases with residents increased from 50% (2 out of 4 cases) before curriculum to 62% (63 out of 99 cases) just after curriculum introduction, and 71% (117 out of 164 cases) at 3 years. RAS use increased in the same time period.	No funding/ 49.5
**EARCS** ^k^ Panteleimonitis 2018 UK Colorectal Surgery Duration not specified [42]	Xi online modules and assessment	VR simulation camera targeting and suturing; EARCS trainer led course with animal and cadaveric models	ERCS Pathway: 2 Case observations; 10 supervised robotic rectal resections, (modular) with video feedback of each case, real-time GAS	Modular training; competence assessed by blind EARCS video assessment	3 surgeons, 1 trainer, two centres (UK, Portugal)	Feasibility and safety of EARCS training pathway/Prospective observational	Compare supervised cases (*n* = 30) vs. independent cases (*n* = 52) after training/ -Perioperative clinical 2015–2017	No difference in operative outcomes with/without proctoring. Significant improvement of GAS^l^ assessment from first 5 to latter 5 supervised cases.	Not reported 74.5
**EARCS** Panteleimonitis 2020 UK Colorectal surgery Duration not specified [43]	See above, details of training pathway not described in detail in this publication				35 expert colorectal surgeons with 7 proctors super- vising all training cases in 26 European Colorectal Units	Short-term outcomes of an international structured training programme /Prospective observational	-Training cases vs. EARCS graduates vs. proctor cases -perioperative clinical data RAS cases from 26 units 2014–2018	No difference in complications, conversion. Significantly shorter operative time + EBL^m^ for proctors. LOS^n^ longer for trainees compared to graduates and proctors	No funding 74.5
**EARCS** Harji et al. 2023 Colorectal surgery France 6 Months [44]	Online modular training	Simulator training, dry lab courses	Bedside assistance console-based training based on EARCS, prior to RAS case components allocated to the different trainees		3 fellows with significant prev. colorectal experience, 6 surgical residents (bedside training only) −52 patients	Feasibility of structured, parallel component-based, training curriculum/Prospective observational	Pre- and post-participant skills assessment/ GEARS, GAS, Perioperative patient outcome data (TME^o^) presented Jan-May 2021	Significant improvement of GEARS 17.3 (95% CI 15.1–21.4) to 23.8 (95% CI 21.6–25.9), p = ROC0.003 for all trainees. GAS component scores improved incrementally for all trainees at each assessment (*p* < 0.001) Rate of post-op complications 21.1% (*n* = 11); median length of stay of 7 (IQR 5–11) days; rate of margin positivity 3.8% (*n* = 2).	No funding 74
**CBC**(Colégio Brasileiro de Cirurgies) Barros et al. 2021 Brazil Observation period 1 year, average time of pre-clinical training was 116 days (range 48–205) [45]	RAS theoretical course, Intuitive surgical, Sunnyvale, USA online course (basics to specialty) - during simulation period Video library via internet, -encourage attendance of 5 specialty cases	VR Simulation; Simulator Simbionix –Endocompany^®^-with RAS nurse teaching -self scheduled -no training with live tissue or animals	Inservice (3 h)- (theoretical and practical training in the platform) 4) Clinical training under mentoring	Company led online assessment, method of assessment before independent operating not specified	39 multispecialty, 5-year experience in specialty, Surgeons 5 year of specialty min, convenience sample of surgeons undergoing RAS training over 1 year	Experience of the first tier of surgeons trained in CBC model/Retrospective observational	Comparing surgeons/Simulation time and score, operative cases March-Dec 2020	20 (51.3%) reached the clinical phase, five (25%) have completed curriculum an operate independent, six surgeons reached target 9 in 90, 3 surgeons participated in 50% of operative cases reviewed	No funding 51
RASC^p^ (Sacrocolpopexy) Catanzarite et al. 2021 USA Gynaecology 3-year Fellowship [46]	Online and in-person da Vinci system training, videos,	Satisfactory completion (scores ≥ 75%) of 22 simulation modules;	Bedside assistance during 3 RASC; console training	Online assessment, Benchmarked operative times for steps, Show safety and efficiency in 5 steps of RASC	7 Gyn (Sarco- colpopexy) fellows, 4 attendings	Examine impact of fellow involvement with RASC/ Prospective observational	Perioperative clinical data with vs. without fellow involvement - operative time and clinical outcome RASC 2012–2018	No significant difference in complications and outcome with/without fellows. No difference in operative times, apart from faster docking time when fellow was involved. Fellows participation significantly improved over time.	No funding 47
Grannan et al. 2021 General surgery USA Residency program (5 years) [47]	Phse1 DaVinci online modules, didactic lectures, journal club, case specific	Phase 2 2 case observations, technical skills, advanced skills simulation	Phase 3 advanced skills min.10 bedside assists Phase 4 Console surgeon cases	Phase completion before progression. Intuitive online assessment of ≥ 80% for Online Training Completion Certificate, 10 bedside cases	43 residents, Year 1–5	Single institution pilot RAS general surgery curriculum /Retrospective observational	Trends over the years resident participation in operations/ curriculum 2016–2019	26 completed RAS basic; 17 completed RAS advanced, 11 equivalency certificates, cases logged increased each year after curriculum institution	Funding not reported 41
Green et al. 2021 General surgery USA During 5-year residency 20–30 months of exposure [48, 49]	Online modules with certificate	2) Da Vinci Dry Lab 3)Console exercises specific docking simulation	3) 10 Bedside procedures 4) 20 Console procedures Unique case-logging system which takes ACT and 6 skills into account	Autonomous progression, standardised progression tracking, - Online module certificates -Docking Proficiency form -Simulation completion sheet -Bedside case logs -Console case logs - console assessment x2 -Extensive case list	Residents	Description of how curriculum was designed, implemented and assessed /Description of curricular elements and evolution over time, Kern’s model	-	Curriculum presented	No funding NA
**Australasian Colorectal pathway** Waters et al. 2021 Australia 12 months [50]	-Case observation -online modules	-Hands on dry lab -simulator practice -Video review and assessment -Robotic console safety course -Animal model robotic course -Robotic accreditation course	- Bedside assist 20–30 cases –postoperative feedback, video review; NTS: formal feedback on- operative technique task management, situational awareness and assistant communication after each case	Industry appointed proctor, logbook maintained by fellows, documenting cart-side assist, component surgeon and primary console surgeon cases throughout; Operative technique, formal video review	Fellows in colorectal surgery	Development and feasibility of fellowship training,/Prospective observational	50 first independent fellow cases vs. 50 consultant cases -fellow logbooks - perioperative clinical data 2018–2020	265 cases with fellow involvement in 2 years (F163,F2 77, F3 75 and F5 50), Independent after case 11, 14, 15 and 12 respectively; no significant difference operative time (*P* = 0.39), blood loss (*P* = 0.41), lymph node harvest (*P* = 0.35), conversion rates (2% versus 4%), anastomotic leaks (1% versus 3%) and R0 resection rates; Clavien-Dindo (III–IV) (10% versus 6%, *P* = 0.25)	No funding 68
Rückbeil et al. 2022 General surgery Germany Duration not specified, study period 2 years [51]	Not specified	40 h simnow VR training, 8 h animal wet lab; NTS: Inter-disciplinary team training in the animal lab	Bedside assistance 20 cases, watch 20 cases on the dual console	Theoretical exam and practical; exam at the animal lab; Debriefing after every case against incomplete steps	3 established general surgeons, 1 RAS mentor	Demonstrate structured RAS implementation and training/ Prospective observational	Perioperative clinical data before RAS introduction and training vs. after, RAS surgeon vs. training surgeons -case load and operative times 2018–2019	Shortened hospital stay compared to before RAS introduction. Operative times longer for trainees. Two trainees complete 8 cases in 2 years; 1 trainee completes 29 cases in two years	Intuitive surgical 40.5
Merriman et al. 2023 Gynaecology USA Duration of Obstetric and Gynaecology (OBGYN), Residency [52]	Intuitive online modules	Orientation: RAS docking lab, safety manoeuvres, VR drills (Morristown protocol	Console practice after case, resident and attending discuss feedback and log case together via the web-based tool	Web-based feedback/case-log progression tracking (resident/attending), Online certificate, 10 bedside cases, Min 20 console cases > 50% of surgeon, letter of verification from program, GEARS assessment; NTS: NOTSS assessment- specific teaching not described	24 residents, 12 for advanced curriculum	Describe a two-phase OBGYN curriculum and feedback tool / Retrospective observational	Pre/post introduction of feedback tool/ GEARS, number of cases operated 2018–2019	Increase of ACGME case entry with introduction of tool, not all cases logged on ACGME were logged into the tool, 12 out of 24 residents initiated advanced phase, 10 completed curriculum, 128 web entries, bedside assistant 96/128 (75%), console surgeon 7/128 (5.5%), both 25/128 (19.5%). 28 console cases: novice 60.7%, intermediate 39.3%, competent 0%	No funding 68
**RoCS** ^q^ Stockheim 2023 General surgery 3 years (Year 3–6 of residency) Germany [53]		Simulator exercises basic and advanced	Bedside assist and procedural steps on second console with increasing complexity NTS: Communication and feedback culture, standardised feedback	Standardise, structured, modular, progressing through levels of competency,90% in simulator exercises, further assessments not provided	12 residents	A feasible and effective training concept for basic competence of trainees /Prospective observational	Pre-post curriculum/ - Trainee participation, -NASA TXL (task load index score) score, -surgical experience score: basic (0–3), advanced, (4–5), expert (6–9) 2017–2021	After six months 52 RAS procedures 82.7% trainee at the bedside and 67.3% participation at 2nd console,107 RAS procedures in 1 year. RoCS, surgeons participated in 78.8% (82/107) as bedside assist. TXL = 26.9 ± 16.6 Frustration (4.8 ± 4.6), Surgical experience score (6.7 ± 4.6)	Open access project DEAL 40.5
**RoCS** Stockheim et al. 2024 Germany General surgery Duration see above [54]					Number of surgeons not outlined, in prev. Publication 12	Examine impact of RoCS on patient outcome/Prospective observational	Before vs. after introduction of RoCS/ Perioperative clinical data 2017–2020	Subgroup analysis by surgical cohort HPB, UGI, CR only. 30-, 90-day morbidity and mortality without change, decreased operative times and LOS for CR, HPB, decrease conversion HPB, decrease. EBL for CR^r^.	Open access project DEAL 49.5
Shu et al. 2023 China Colorectal Duration not specified[55]	Virtual learning tests	On-site simulations and animal experiments	Proctoring during one case	Structured training, VR tests, After trainee completes first robotic surgery independently qualification of RAS colorectal surgery is issued	10 surgeons; 5 with extensive laparoscopic experience (300–500 cases) 5 surgeons with less experience (50–100 prev. lap. colorectal cases)	Evaluate RAS curricula in China for surgeons with different laparoscopic experiences /Retrospective review	Surgeons with vs. surgeons without previous laparoscopic experience/ Perioperative clinical data first 15 independent cases	Significantly shorter operative time, less blood loss, shorter hospital stay, complication rate (intra and postoperative), reoperation rate and cost for surgeons with extensive prev. laparoscopic experience.	Natural Science Foundation of Shanghai; 50
Unruh et al. 2023 USA General surgery during 5-year residency [56]	Online modules, hands on intuitive service training	SimNow VR simulations (16 modules) throughout junior and senior residency phase	10 bedside assists, stepwise increase of console experience, intraoperative, video review,	Progression through curriculum phases, Simulation > 90%, Verbal feedback intraoperative, video review, SIMPL evaluation app, GEARS, equivalency certificate 20 operations of min 50%	25 residents	Curriculum description and characterise immediate impact on residents /Retrospective observational	Pre-post curriculum/ Operations logged, resident participation Tracking of ACGME case-logs 2017–2021	681 operations logged by 25 residents in 4 years. Bedside experience largely PGY1. PGY 4: mean 9.1 ± 7.7 console operations per resident, PGY 5: mean 12.0 ± 4.8 console operations per resident; robotic certification increased among graduating residents 0% 2013, 50% 2014, 80% 2015, and 100% for 2016 and 2017.	No funding 64.5
Haghshenas et al. 2023 USA Acute care Surgery (ACS) Duration of ACS Fellowship [57]	Fundamentals of Robotic surgery (online modules)	-Skills and procedure simulations on RAS practice console -VR exercises -Hands-on boot camp docking simulation, NTS: -crisis training, - team training All with simulation	OR set-up, min 10 cases bedside, schedule cases from clinic under supervision, reach proficiency in specific operative steps	Gain proficiency through curriculum phases, -intake assessment -score min 80% on FRS and on advanced Simulation, plan and execute room layout, min 10 bedside cases, complete 15 cases > 50%	Acute care surgery fellows	Description of RAS ACS curriculum / Description of department guidelines and outline of curriculum	No examination of curriculum is undertaken in this publication	Curriculum presented	No funding NA
**Portsmouth perspective** Portsmouth is also an ESCP^s^ host centre Piozzi 2024 UK Colorectal surgery Operative fellowship phase 6–12 months [58]	9 cardinal pillars of theoretical training, ROBO-CERT/Robotic Driving Licence course (ALSGBI)	VR and Dry Lab as part of the ROBO-CERT course 50 h; NTS: as part of the ROBO-CERT course: “soft skills, such as clear communication between the console surgeon and the bedside team”	Case observations on 2nd Console or on SCOPEYE, bedside assist, RAS hands-on fellowship, increasing case complexity 1–3, RAS immersion course 1week, operative fellowship with video review after every case, RAS TME index case	Modular operative training, Operative fellowship only after 50 h of Simulations with documented level of performance, basic robotics exposure (wet lab), laparoscopic colorectal surgical proficiency (local evaluation and logbook), video assessment and feedback after every case	Colorectal surgeons with documented colorectal laparoscopic proficiency	Description of ROBO-CERT Course and 6–12 months colorectal TME fellowship / Description of courses and Modules used in the RAS colorectal training in Portsmouth,	See below	See below	No funding NA
**Portsmouth perspective** Stefan 2024 Colorectal UK Duration see above [59]				Modular operative training	Four trainees (97 cases July 2015-dec 2017)) vs. 3 trained colorectal surgeons (80 cases May 2013-June 2015)	Compare perioperative and oncological RAS rectal TME outcomes in a modular training program/Retrospective review on prospective database	Consultants only versus supervised trainees/ perioperative clinical outcomes 2013–2017	No difference was observed between the 2 groups in terms of R0 resection rate, number of lymph nodes, conversion rate, and perioperative and oncological outcomes (local recurrence, distant metastasis, and 1-, 3-, and 5-year overall and disease-free survival), 25-minute longer operating times with surgical trainees	No funding 46.5
Ismail et al. 2020 BIARGS Delphi UK Gynaecology During postgraduate Gynaecology training [60]	E- learning	Simulation. NTS: Non-technical skills mentioned in text, not part of the skills list	Case observation, min 15 cases supervised, competency based operative training	Competency based curriculum, Curriculum is a list of skills to be assessed by surgical mentor in 3 skill levels; 3 modules (assist, surgeon skills, continued audit)	No- assessment of curriculum	Delphi consensus study for curriculum development	-	-	BIARGS NA
RoSTraC^t^ Thomaschewski 2024 General/Visceral Surgery Germany 12–18 months [61]	VR lectures, Video library, theoretical basics on RAS	VR simulation (40 h), Dry lab training on Lübeck toolbox, complex skills on synthetic organs	30 RAS observation, 40 assists, procedural steps to full procedure	Standardise, proficiency goal directed, stepwise. Video evaluation of dry-lab Gastroenterostomy using GEARS by training centre, intraoperative feedback from trainer	General surgical residents 7 surgical residents in 5 RAS centres nationwide	Feasibility of curriculum and transferability of acquired skills / Prospective observational pre-post-test one group	Participants skills pre-and post-curriculum/ Curriculum engagement, GEARS score	Curriculum completed by all, VR: median 3 repetitions (range 1–9) to meet targets, median of 11 repetitions (5–15) for anastomosis task, significant improvement in GEARS on Anastomosis, median of 12 (range 5–21) RAS procedures on the console	Project DEAL; Intuitive surgical, Sunnyvale, CA, USA /59.5
Gauci et al. 2024 Australian private sector RAS colorectal training 12 months [62]	Online intuitive modules	VR simulation score > 90%; NTS: ability to troubleshoot	Bedside assisting, operative training along specific modular steps, video review	Transition through curricular phases, Modular training, Complete advanced course in the transition to mastery, min 20 Cases (35 cases/year)	Conventionally trained colorectal surgeons	Expand RAS expertise in the private health care sector in Australia/Description of program only	Planning to audit case series	3 fellows completed program	Open access funding by CAUL /NA
Yeung2025 Colorectal USA 1 year apprenticeship, 9 individual months with different attendings + 3 months project [63]	Orientation lead by RAS PA, online intuitive certificates, case and attending specific videos	RAS VR Simulator score 85%, dry lab (docking etc.), wet lab	Component based console training, specific intraoperative feedback, debriefing, Video review	Regular, standardised proficiency progression tracking, Completion of all parts of curriculum summative assessment by attendings	3 international advanced colorectal oncology fellows	Feasibility of structured RAS apprenticeship training program,/Prospective observational, pre-post self- assessment	Compare fellows self-assessed GEARS and GAS pre/post fellowship, present patient outcome 2023–2024	64–98 console cases/fellow, RAS right colectomies (13–26), RAS high anterior resections (13–17), RAS low anterior resections (23–30); All significant improvements in self-assessed GEARS score, mean score of 12 to mean score of 27.2, *p* = 0.0065; modified GAS: significant improvement; RAS right hemicolectomies (*p* = 0.0052) and RAS anterior resections (*p* = 0.0005)	No funding/ 73
Englert 2025 General surgery Publication in German 2 years [64]	Hospital specific video library, + freely available video library	Structured simulation- self-guided	Tableside assistance (initially with help), stepwise console training, increasing case complexity (I-III)	Assessments not specified in report, move through modules	6 surgeons, 3 general (visceral) surgical trainees, 3 specialists	Description of curriculum for residents /Description of program only	Evaluation by questionnaire, methods do not describe the collection of data in detail	1 specialist completed program and operates independently, 100 teaching operations since 2022, docking time reduced from around 40 min to 20 min in the first 100 interventions,	Not declared/33.5

a ACGME Accreditation Council for Graduate Medical Education, b EAU/ERUS European Association of Urology/EAU Robotic Urology Section, c RARP Robotic Assisted Radical Prostatectomy, d UPMC CGSO University of Pittsburgh Medical Centre Complex general Surgery Oncology, e UGI Upper gastrointestinal surgery, f RPD Robotic Pancreatoduodenectomy g HPB hepatobiliary surgery h Non-UPM: (UAB = University of Alabama at Birmingham, CU = University of Colorado Anschutz Medical Campus, JHU = Johns Hopkins University, UC = University of California, WashU = Washington University in St. Louis), i SERGS Society of European Gynaecologic surgery, j APDCRS Association for Program Directors in Colon and Rectal Surgery, k EARCS European Academy of Robotic Colorectal Surgery, l GAS Global Assessment Score, m EBL estimated blood loss, n LOS length of stay, o TME Total Mesorectal Excision, p RASC Robotic Assisted Sacrocolpopexy, q RoCS Robotic Curriculum for young Surgeons,

r CR Colorectal surgery s ESCP European Society for Coloproctology, t RoSTraC Robotic Surgery Training Curriculum

## Results

We included 39 publications regarding 29 comprehensive RAS curricula in our review. Table 2 All the publications were from developed countries; USA(*n* = 20), Europe *n* = 15 (European Societies (*n* = 3), UK (*n* = 5), Germany (*n* = 5), France (*n* = 1) Netherlands (*n* = 1)), Brazil (*n* = 1) Australia (*n* = 2), China   (*n* = 1).

The participants in the curricula studies included surgical trainees (*n* = 16), surgical fellows (after obtaining Certificate of Completion of Training (CCT))  (*n* = 6), established surgeons (*n* = 6) and multi-skill levels (*n* = 2).

There were multiple publications on the European Association of Urology (EAU) Robotic Urology Section(ERUS) curriculum (*n* = 2) [19, 28], the University of Pittsburgh Medical Centre (UPMC) curriculum (*n* = 7) [30, 32–37], European Academy of Robotic Colorectal Surgery (EARCS) curriculum (*n* = 3) [42, 43, 58], Robotic Curriculum for young Surgeons (RoCS)(*n* = 2) [53, 54] and the colorectal Portsmouth curriculum (*n* = 2) [58, 59]. 

The curricula included the specialties of urology (*n* = 2), general surgery (*n* = 12), upper GI (Gastrointestinal) surgery (*n* = 1), colorectal (*n* = 7)), gynaecology (*n* = 5), thoracic surgery (*n* = 1) and acute care surgery (*n* = 1).

Overall, the quality of the studies was low (MMERSQI score in Table 2). None of the publications used randomisation. Most studies were observational, often including one group of trainees with a post-training examination design. None of the studies performed power calculations. The statistics employed were mostly simple descriptive and statistician involvement was not mentioned in any publication. Participant characteristics (*n* = 20) [25, 26, 29, 32, 39–43, 45–47, 51–56, 59, 64]and validity of participant assessments were often(*n* = 17) not reported [24–26, 29, 37, 39, 41, 45–47, 50, 51, 53–55, 59, 64]. Participant number in publications were low ranging from 1 to 43 with a total of 418 participants in 39 publications. No long-term patient outcome data were available.

## Curricula elements

We have included a summary of the curricula elements used in the analysed publications in Table 3. Many curricula (*n* = 15) included industry provided elements. Intuitive surgical, Sunnyvale, CA, USA, online modules, ‘Inservice’ teaching, VR simulation, wet lab courses and equivalency certificates were all mentioned. (See Table 2) [24, 26, 29, 39–42, 45–47, 50, 52, 56, 62, 63].

All curricula included simulation; virtual reality (VR) (*n* = 16), dry lab (*n* = 11), wet lab/bio-tissue/cadaveric (*n* = 12) with multiple curricula including more than one modality. Some curricula (*n* = 3) included specific docking/ergonomic simulation training additionally to any manufacturer provided in-service training [48, 52, 57]. Three curricula required a minimum VR simulation duration, varying from 10 to 40 h [29, 51, 58]. Other curricula (*n* = 9) specified simulator proficiency scores, varying from 75%- >90% [32, 39–41, 46, 53, 57, 62, 63]. Eight curricula provided simulation through attendance of courses [19, 28, 38, 39, 42, 57–59].

Online modules were principally used for the delivery of theoretical knowledge. Only a few curricula (*n* = 4) specifically mentioned e-learning content additional to industry-developed online teaching [15, 19, 27, 34]. A few publications mentioned a video library (*n* = 4) hospital or teacher specific [27, 31, 40, 45]. Two curricula included additional reading assignments for their trainees [29, 47]. 

Most curricula (*n* = 18) included bedside assists (2–50 cases, most commonly 10 cases) and less commonly, case observations (*n* = 7), in their operative training [24, 25, 29, 41, 42, 44, 46, 48, 50, 51, 53, 60–62, 64]. Curricula from Germany required a comparatively higher number of bedside assists (20–40) with additional console observations (20–30 cases) compared to other countries [51, 61]. Englert et al. focussed 3 steps of their 6-step program on teaching tableside assisting and one additional step on console observation [64]. 

Console teaching was described as stepwise (*n* = 11), modular (*n* = 3), component based (*n* = 1) and with increasing difficulty from moderate to complex (*n* = 4) [19, 24, 28, 39, 40, 42, 46, 53, 56, 61, 62, 64]. The number of console cases mentioned (*n* = 7) varied from 1 to 30 cases [25, 41, 42, 48, 55–57]. Several (*n* = 5) curricula specified a percentage of console time (usually 50%) for cases to be counted towards progression [39, 52, 56, 57, 65]. Video review was mentioned as a feedback technique for operative teaching in several curricula (*n* = 7) [30, 42, 50, 56, 58, 62, 63]. 

Non-technical skills (NTS) were mentioned in 14 curricula. NTS teaching was part of e-learning, simulation or operative training. NTS training included emergency scenarios/team training (*n* = 2), situational awareness (*n* = 1), communication (*n* = 2), troubleshooting (*n* = 4), decision-making (*n* = 1) and assessments (*n* = 4) [25, 29, 38, 39, 41, 50–53]. Moit et al. and Rusch et al. included NTS assessments as part of formalised mentorship feedback in operative training, however, surgical mentors were unfamiliar with the assessment or score and did not provide written NTS feedback as indicated in the curriculum [38, 40]. 

Descriptions of curricula mentioned proficiency, competency or mastery based training, often using the terms interchangeably [19, 26, 28, 30, 34–36, 40, 44, 45, 48, 50, 52, 56–59, 61–63]. Gauci et al. specific for proficiency being a higher skill level than mere competency [62]. The assessment methods included were video review (*n* = 5) [19, 28, 30, 38, 42, 58, 61, 62], logbook evaluation (*n* = 4) [29, 38, 50, 58] and standardised evaluation by mentors *n* = 8 [19, 25, 40, 41, 48, 52, 56, 63]. In several publications, the method of final curriculum assessment remained unclear [24, 26, 29, 37, 45–47, 51, 53, 55, 64]. 

**Table 3:** Table of curricula content in the examined publications (Number in brackets specifies how many of the 29 examined curricula include the element)

Theory	Simulation	Operative practice	Progression and Assessment
• E-learning o manufacturer provided (17) o additional e-learning content (4) • Intuitive surgical, Sunnyvale, USA course (2) • Video Library (4) • Lectures/tutorials (4) • Reading assignments (3) • Surgeon led Webinars (1) • In-Service training with a manufacturer representative (4) • Ergonomic course additional to industry provided training (3)	**Modality** • VR Simulation (16) • Dry Lab Simulation (11) • Wet Lab Simulation (12) **Progression** • Time based 10–40 h (3) • Minimum number of 10 attempts (1) • Proficiency based progression 75%−90% (9) • Ongoing simulation during operative phase (1)	• Modular training to predefined benchmarks of proficiency with increasing complexity of operative steps (6) • Video review for teaching and feedback (6) • Formative and summative assessments using standardised scores (11)	• Proficiency/competency/mastery progression (16) • No summative assessment mentioned/time spent in program (12) • Operative logbook case number 1–30 (7) • Minimum percentage of console time (50%) (5) • Number of formalised mentor assessments (e.g. 25 cases operated with 5 summative mentor assessments) (2) • Anonymous external video review with validated operative skills score (3)

## Outcomes

The outcomes reported in the publications included Kirkpatrick’s level 1 self-reported outcomes (*n* = 2) level 2 (evidence of learning), skill increase (*n* =5) level 3 (change in clinical behaviour), operative volume (*n* = 13), and intraoperative skill measurement (*n* = 6) as well as level 4 short-term clinical outcomes (*n* = 12) with some publications reporting more than one level of outcomes. (Table 1) Due to the heterogeneity of the intervention and comparator in the studies, a metanalysis was not attempted.

Level 1: Two studies presented trainee self- reported data. The self-reported data consisted of completing the curriculum and clinical involvement [29] and a case-log system reporting operative steps and percentage of active console time [39]. 

Level 2: Skill assessment in simulation was undertaken (*n* = 5) in low fidelity simulation (VR) [25, 32] and high-fidelity simulation [33, 36, 61]. All studies showed an improvement with engagement in the curriculum.

Level 3: Clinical behaviour was reported in several studies (*n* = 18). Earlier studies reported the “uptake” of RAS after curriculum completion, comparing themselves favourably to other teaching methods of the time [24, 26, 30]. Mirheydar found the number of proctored cases varied widely for surgeons of different experience levels (5 to 40 cases). Trainees received more extensive proctoring until they were able to perform RAS independently [24]. The RAS operative volume of trainees was found to increase with the implementation of a curriculum [30, 41, 47, 56]. Other studies just presented trainee caseload [45, 51, 52] or stated how many teaching operations have been completed generally, demonstrating an overall reduced docking time with introduction of the curriculum [46, 53, 54, 64].

Several (*n* = 6) studies assessed the changed clinical behaviour using intraoperative scores GEARS, RARP (Robotic Assisted Radical Prostatectomy) procedure score, OSATS and GAS. An improvement in RAS intraoperative skills related to trainee engagement with the curriculum was found in all studies which presented skill assessments [19, 38, 40, 42, 44, 63]. 

Level 4: Studies examining short-term clinical outcomes (*n* = 12) examined, operative time, estimated blood loss (EBL), conversion rates, complication rates, length of stay and operative oncological quality measures (R0-resection, margin, lymph node yields). The comparators varied [26, 28, 34, 35, 37, 42–44, 46, 50, 55, 59]. Several studies (*n* = 4) found significantly longer operative times in trainees compared to their proctors, with other perioperative quality measures showing no significant difference [28, 42, 51, 59]. Panteleimonitis et al. also found proctors had less blood loss and length of stay than recent graduates or trainees [29]. Others (*n* = 2) found no difference in outcomes [46, 50]. Three publications presented patient outcomes without comparator [26, 37, 44]. 

Two publications, examining the UPMC curriculum, compared patient outcome data from surgeons who had completed the RAS curriculum to surgeons who had not [34, 35]. Tam et al. found surgeons, that had self-selected to complete the RAS hernia curriculum, had a significantly shorter operative time and less procedural cost compared to those who had not. They found that non-trainees had to “practise” on approximately 28 live hernia cases until they matched the operative time of curriculum graduates in their first case [34]. Rice et al. compared perioperative outcomes from three generations of surgeons, early adopters, surgeons who received mentoring from early adopters and surgeons, who had received the structured proficiency-based curriculum after 2013. Subsequent generations of surgeons showed improved operative times, conversions, lymph-node yield and length of stay without a significant difference in complication rate (pancreatic fistula) [35]. 

Shu et al. compared surgeons with and without extensive previous laparoscopic experience (300–500 cases) who underwent the RAS training curriculum in China. Surgeons without extensive laparoscopic experience (50–100 cases) had significantly longer operative times, more complications, blood-loss, re-operations and longer hospital stay, leading them to conclude that the curriculum was insufficient for surgeons without extensive laparoscopic experience [55]. 

## Discussion

**Fig. 3:**
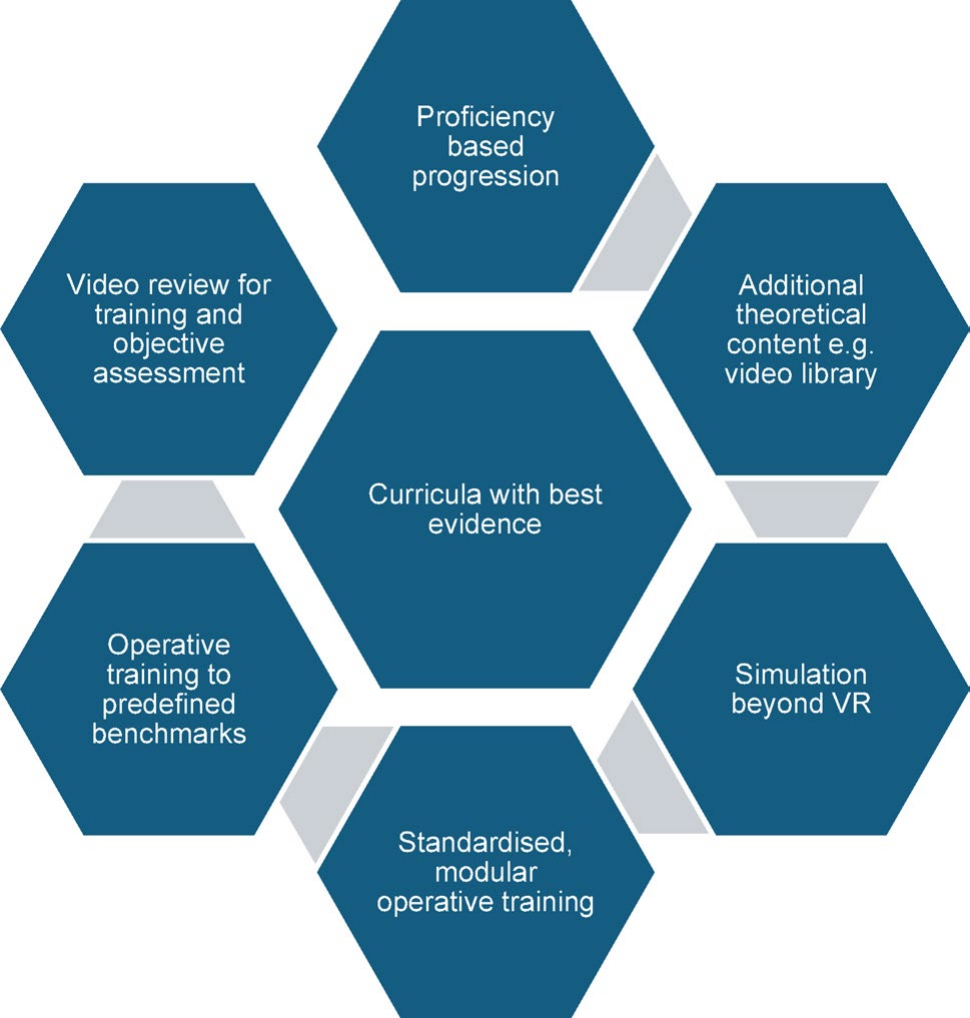
Common characteristics of curricula with best evidence presented

We aimed to systematically review previously published comprehensive RAS curricula. The main finding from this review was that there were clear benefits to training from the development of a standardised RAS curriculum that includes simulation. A curricula framework based in current educational pedagogy also appears to be advantageous. The examined curricula varied in the use of simulation modalities, intra-operative training, assessment and non-technical skills training. The recent increase in publications on RAS curricula for surgical trainees demonstrates that there is interest in a standardised training curriculum. See Fig. 3.

The introduction of a RAS curriculum was found to be beneficial in all the studies. It led to increased RAS operative volume for trainees [30, 41, 47, 52, 56],. Additionally, skill measurements improved in trainees who engaged with the curriculum [19, 25, 32, 33, 38, 40, 42, 44, 61, 63]. Operative time was the only parameter that was significantly different (longer) for trainees compared to their trainers [28, 43, 51, 59]. At UPMC a curriculum was developed for RAS pancreaticoduodenectomy (PD), a complex procedure with limited live training opportunities. The RAS PD curriculum showed a clear benefit compared to mentoring alone [35]. An adapted UPMC curriculum for RAS hernia surgery showed an impressive difference in perioperative outcomes of graduates vs. non-graduates for the much simpler procedure [34]. This evidence translates to an ethical obligation for surgeons and surgical teachers to provide and enforce a well-structured, standardised curriculum, for complex operating like RAS.

All the comprehensive RAS curricula included simulation training, which has a good evidence base for improving outcomes [66–69]. This included curricula from countries with a strong tradition of apprenticeship training [51, 53, 61, 64]. This shows that simulation has evolved from a ’nice to have’ in the open and laparoscopic era to an essential component of RAS training. However, the utilised simulation training varied widely amongst the curricula, from time-based VR simulation without specific assessment, to validated complex proficiency-based simulation curricula with procedure-specific elements using cadaver, animals or bio tissue. The most convincing benefit is shown with proficiency-based curricula, providing trainees with benchmarks to be achieved in simulation or operative practice [19, 28, 35, 38, 42, 50, 58, 59]. This reinforces the role of simulation as an important curriculum component and is most effective when combined with a benchmarked proficiency-based assessment.

Several curricula have been developed by surgical societies, for example, ERUS, Society of European Gynaecologic surgery (SERGS), EARCS [19, 28, 38, 42, 59, 60]. These incorporate evidence-based knowledge training [70] and simulation training [6, 71] in addition to educational content provided by industry. The curricula commonly included standardised proficiency-based mentorship training with predefined benchmarked modular steps according to level of difficulty. Learning was demonstrated by the completion of an increasing number of operative steps by the trainee. Assessment and credentialing were undertaken with the use of validated assessment tools (e.g. GEARS, GAS, RARP Score) [19, 28, 38, 42, 44] and video review for final curriculum assessment.

Locally developed curricula, such as the UPMC curriculum and the Portsmouth curriculum, follow a similar structure, with proficiency-based simulation courses, the use of video review, and structured mentoring or a minimum percentage (> 50%) of active time for console cases [30, 39, 42, 50, 56, 58, 59, 62, 63]. 

The mode and conduct of education delivery can be linked to the outcomes. The inclusion of performance benchmarks for feedback and assessment as well as structured, modular intraoperative training were linked to good evidence of skill improvement and satisfactory short term patient outcomes. The consistent inclusion of simulation training and online models in all curricula and recurrently mentioned ‘in-service’ training is possibly driven by industry requirements [72]. Shu et al. found their curriculum to be inadequate for surgeons without extensive laparoscopic experience. This could be due to inadequate simulation training or the provision of limited mentoring (proctoring). The curriculum they describe is not unlike current RAS training across Europe [55]. 

RAS provides unique non-technical skill (NTS) challenges to the surgeon, especially in situational awareness and communication [73, 74]. Few curricula require NTS training beyond industry ‘troubleshooting ‘. Introducing specific NTS training, including emergency conversion, RAS specific situational awareness (what happens off screen) and communication into RAS curricula could improve safety and enable young surgeons to anticipate common pitfalls unique to this modality [49, 75–78]. Future RAS curricula should include NTS to make surgeons aware of the easily avoidable pitfalls, reduce errors and increase patient safety.

Many of the assessed studies did not describe the method they used for curriculum assessment nor its validity, weakening the educational evidence. The transition to independent operating on the RAS console often remained at the discretion of a single mentor or case number, introducing bias and risk compared to an external assessment method.(see Table 1) The implementation of recently emerging objective assessment metrics [79] and usage of a common standardised credentialing pathway [80]for RAS could serve as a safety measure for baseline skill. Objective, validated assessment methods improve bias, reduce risk and make curricula comparison and improvement (using Kern ‘s steps 6) possible.

The main strength of this systematic review is the breadth of search, giving us the opportunity to consider a wide variety of RAS educational literature. To our knowledge this is the first review comparing comprehensive curricular pathways for RAS training. The analysis of comparable publications relevant to operative skills intends to provide surgical educators with actionable understanding of the available curricular pathways. An inherent weakness of this review is the low quality of available evidence. Only a few of the publications regarding the 29 curricula provide Kirkpatrick level 3 (skill) and 4 (clinical outcomes) of evidence. All publications have significant limitations.

A future RAS curriculum should be standardised and based on available evidence of best psychomotor learning. This will include simulation with feedback and proficiency-based progression, enabling deliberate practice before live operating. Intraoperative mentoring should be adapted to the previous skill and experience of the surgeon. Modular training outlines a clear training path and ensures trainee progression while maintaining patient safety. Progression can be tracked by intraoperative performance metrics, which can be incorporated into objective feedback. Objective, unbiased skill assessments ensure patient safety and serve as a tool for curriculum evaluation. The surgical robot reduces the burden of progression tracking and video recording, which can be used for feedback and assessment. The implementation of curriculum elements outlined by surgical societies appears to be desirable and feasible. Surgical societies have already validated and implemented their published curricula on a small scale. Further evidence supporting RAS curricula, the benefit of content and components, the method of curriculum assessment and its validity must be included in future educational research alongside robust statistics. Patient outcome data, including long-term outcomes, will deliver the most robust curricula evidence and are necessary to justify the considerable cost and effort associated with RAS curriculum implementation.

## Conclusion

Multiple comprehensive RAS curricula pathways facilitating surgeons’ safe progression to operative independence have been developed. However, there is heterogeneity in the quality of training and assessment methods. The overall level of evidence is low since few studies examine participants skill and patient specific outcomes and all the studies have significant methodological limitations. Despite this the curricula do share important common elements, like simulation training. Several surgical societies have published curricula integrating specialty specific knowledge and simulation elements, structured mentoring pathways and objective assessment tools. Important RAS specific non-technical skills remain underrepresented in current published curricula. The available body of evidence underscores the feasibility and benefit of standardised RAS curricula for surgical trainees. Future research should consider comparing the short- and long-term clinical outcomes of surgeons who have completed RAS curricula compared with their peers.

## Supplementary Information

Below is the link to the electronic supplementary material.

Supplementary Material 1

Supplementary Material 2

## Data Availability

No datasets were generated or analysed during the current study.
